# Bioenergetic Profiling in Glioblastoma Multiforme Patients with Different Clinical Outcomes

**DOI:** 10.3390/metabo13030362

**Published:** 2023-02-28

**Authors:** Vivi Bafiti, Sotiris Ouzounis, Eleni Siapi, Ioanna Maria Grypari, Andreas Theofanopoulos, Vasilios Panagiotopoulos, Vasiliki Zolota, Dimitrios Kardamakis, Theodora Katsila

**Affiliations:** 1 Institute of Chemical Biology, National Hellenic Research Foundation, 11635 Athens, Greece; 2 Department of Pathology, School of Medicine, University of Patras, 26504 Patras, Greece; 3 Department of Neurosurgery, University Hospital of Patras, 26504 Patras, Greece; 4 Department of Radiation Oncology, University of Patras Medical School, 26504 Patras, Greece

**Keywords:** untargeted metabolomics, glioblastoma multiforme, translational biomarkers, bioenergetics, oncometabolism, metabolic reprogramming, epigenetic modulators, drug repurposing

## Abstract

The accumulation of cell biomass is associated with dramatically increased bioenergetic and biosynthetic demand. Metabolic reprogramming, once thought as an epiphenomenon, currently relates to disease progression, also in response to extracellular fate-decisive signals. Glioblastoma multiforme patients often suffer misdiagnosis, short survival time, low quality of life, and poor disease management options. Today, tumor genetic testing and histological analysis guide diagnosis and treatment. We and others appreciate that metabolites complement translational biomarkers and molecular signatures in disease profiling and phenotyping. Herein, we coupled a mixed-methods content analysis to a mass spectrometry-based untargeted metabolomic analysis on plasma samples from glioblastoma multiforme patients to delineate the role of metabolic remodeling in biological plasticity and, hence, disease severity. Following data processing and analysis, we established a bioenergetic profile coordinated by the mitochondrial function and redox state, lipids, and energy substrates. Our findings show that epigenetic modulators are key players in glioblastoma multiforme cell metabolism, in particular when microRNAs are considered. We propose that biological plasticity in glioblastoma multiforme is a mechanism of adaptation and resistance to treatment which is eloquently revealed by bioenergetics.

## 1. Introduction

Glioblastoma multiforme (GBM), a World Health Organization (WHO) grade IV central nervous system (CNS) tumor, is characterized by intra-tumoral heterogeneity and inter-individual variability, which are key determinants of disease progression and low survival rates (average survival time of 12–15 months) [1,2,3]. The current—and since almost two decades—first line standard of care for GBM includes surgical resection followed by adjuvant, fractionated radiotherapy (RT) with concomitant and maintenance temozolomide (TMZ) chemotherapy [3,4,5]. Diagnosis and recurrence monitoring are routinely carried out by computed tomography and magnetic resonance imaging modalities, yet their application is limited when it comes to large-scale screening due to radiation side effects, cost, and, sometimes, inaccessibility. Alternatives of clinical utility and, at the same time, clinical validity are missing as tumor biopsy-based strategies (such as fine needle aspiration biopsy and genetic profiling or sequencing of circulating tumor DNA) are themselves too invasive in nature for repeated sampling [6,7]. Liquid biopsy-methods are emerging as they are accompanied by technological advances in both dry- and wet-lab approaches [8,9]; however, they are not ready for prime-time, when the unmet needs are listed either for the general public or GBM patients.

Metabolomics has come forward as a prevailing analytical strategy that maps holistically the molecular status in a given biological sample informing about the interplay of the genome and environmental influences. Such individual profiles (metabotypes) serve as snapshots, which are extremely informative in nature as they are the net result of tumor and host biology in the presence of xenobiotics. Therefore, metabolomics datasets not only allow for hypothesis-driven data interpretation, but also the generation of hypotheses. Of note, metabotypes may complement other omics data layers (metabolomics-based multi-omics) and/or predict response to treatment via mathematical modeling (pharmacometabolomics).

Metabolic reprogramming, a hallmark of cancer, is involved in tumor aggressiveness and treatment resistance allowing for tumor cells to adapt to their bioenergetic and biosynthetic demands [10]. In GBM, tumors appear to manipulate and exploit normal brain cells, affecting almost all cell types at the tumor niche via intracellular biological plasticity and multiple types of communication [11]. Thus, we and others map the genome-environment interplay with high time sensitivity and spatial resolution via multi-omics as a key strategy for precision oncology [12,13].

Efforts to map the GBM metabolic landscape are multi-level (tumor, tumor niche, biofluids). Key cell metabolism alterations in GBM relate to disordered lipid metabolism, dysfunctional oxidative phosphorylation, and increased Warburg effect [14,15]. Recently, the role of epigenetic modulators as regulators in tumor cell metabolism has gained great interest. Such epigenetic modulators include histone modifications, DNA methylation, nucleosome remodeling, and non-coding RNAs (long-noncoding RNAs, circular RNAs, microRNAs) [16,17]. In particular, microRNAs act as regulators of metabolic gene expression either directly or by regulating metabolism-associated oncogenic signaling pathways, oncogenes, or tumor suppressors. In gliomas, microRNAs have been reported to target mRNAs of enzymes that participate in glycolysis, oxidative phosphorylation, lipid metabolism, and mitochondrial energy metabolism as well as glutamine metabolism [18,19]. To name but a few, Alfardus et al. reported miR-619-5p, miR-4440, and miR-4793-3p regulating lipid metabolic pathways in GBM [20], while Kwak et al. identified miR-3189 and its role in glucose metabolism by targeting GLUT3 in GBM cell lines [21]. Oncogenic K-Ras, EGFR, c-myc, and mTORC2 as well as PI3K/Akt and LKB1-AMPK pathways are also regulated by microRNAs [14].

Up to now, only a limited number of studies have explored the metabolic landscape of GBM patients focusing on biofluids in line with the anticipation that the latter will pave the way towards non-invasive procedures in the clinic, despite the advantage of untargeted mass spectrometry-based metabolomics to detect as many metabolites as possible at once, identify unexpected metabolic alterations, and characterize novel metabolites in biological samples [12,22,23,24,25]. Even fewer are the datasets that are well-balanced both at the exploratory and validation phases [26,27] coupling untargeted metabolomics to a mixed-methods content analysis.

Herein, we designed, employed, and optimized a strategy coupling a mixed-methods content analysis (i.e., gold standard approach for content analysis) to a metabotype approach for GBM patients to delineate the role of metabolic remodeling in biological plasticity and, hence, disease severity. For this, GBM bioenergetic profiles were explored at the time of diagnosis and during follow-up (GBM patients, n = 21; plasma samples, n = 122). Next, the informative relationships through which metabolites are connected were interrogated. This holistic strategy presents a great opportunity to unveil patterns and provide new insights for GBM biology and drug repurposing.

## 2. Materials and Methods

### 2.1. Mixed-Methods Content Analysis

We employed a mixed-methods content analysis, a gold standard approach for a content analysis that consists of deductive (quantitative) and inductive (qualitative) phases, while contemporary definitions are considered. For data and text mining as well as data analysis, peer-reviewed literature, omics datasets, and clinical trial outcomes (as of 2022) were mined to interrogate GBM plasma metabotypes. We have also developed a novel framework to meet our analytical demands, exploring data (both context and content). Literature data from Scopus and PubMed/MEDLINE were queried. Scopus and PubMed/MEDLINE are the largest citation and abstract databases of peer-reviewed literature. To account for selection biases, private and publicly available texts have been assessed (based on the inclusion/exclusion criteria set). Keywords and MeSH terms (www.nlm.nih.gov/mesh, accessed on 23 January 2020) included “GBM OR glioblastoma AND plasma AND metabolomics”, “GBM OR glioblastoma AND untargeted metabolomics”, and “GBM OR glioblastoma AND metabotypes”. We questioned the interim output further for open data (yes/no), sample size (validated by a power analysis), research approach, and publication impact/metrics. Studies that failed to meet inclusion criteria or studies on non-human samples were excluded. Two co-authors (V.B. and T.K.) co-analyzed the interim and final outputs, and then, the percentage of inter-rater agreement was calculated. To account for biases, Cohen’s kappa statistic and percentage agreement were also determined with multi-categorical ratings.

To interrogate further GBM plasma metabotypes as well as the informative relationships through which metabolites are connected, we queried the Human Metabolome Database (HMDB) [28], the Metabolomics Workbench, https://www.metabolomicsworkbench.org/, (accessed on 9 October 2020, 9 October 2021 and 29 December 2022), and Metabolights [29].

### 2.2. Clnical Cohort and Samples

The study protocol is in accordance with the Declaration of Helsinki and has been approved by the ethics review board of the General University Hospital of Patras, Greece. IRB protocol number: 8735/142. Study participants signed a written informed consent. Patients (n = 21) have received the diagnosis of GBM based on the WHO criteria applicable at the time of recruitment (according to the WHO classifications of 2016 and 2021), and the standard-of-care treatment protocol was applied [3,30]. Overall survival (OS) was determined from the time of diagnosis until death or last follow-up (12 months). Clinical and demographic characteristics are shown in Table S1.

### 2.3. Untargeted Metabolomics

#### 2.3.1. Sample Preparation

Sample collection, processing, and storage for both blood and plasma samples (n = 122) were performed as described in Chalikiopoulou et al. [31]. For LC-MS-based untargeted metabolomics analysis, plasma samples were thawed on ice at 4 °C, and 400 μL of plasma were aliquoted into a 2.0 mL low-adherence microcentrifuge tube. Sample extraction was carried out by ice-cold methanol (3:1) (*v*/*v*) for best metabolite yield, and then, the mixture was vortexed for 15 s. Samples were centrifuged at 15,800× *g* for 15 min at room temperature to pellet the protein precipitate. The supernatant was transferred into a new 1.5 mL low-adherence microcentrifuge tube and dried down (lyophilized) in a centrifugal vacuum evaporator for 18 h. No heating was applied during the drying process. Next, samples were reconstituted with 180 μL 80:20 methanol in water (*v*/*v*), sonicated for 10 min, and centrifuged for 1 min at 14,000× *g* (room temperature). The 100 μL-aliquots were transferred to each insert of liquid chromatography (LC) glass vial, N8. Quality control (QC) and internal standard (IS) samples were prepared as described in Chalikiopoulou et al. [31].

#### 2.3.2. Chromatographic Conditions

The liquid chromatography separation was performed with an Accela ultra-high-performance LC (UHPLC) system. A polymeric SeQuant^®^ ZIC^®^-pHILIC column (5 μm, 150 mm × 2.1 mm) (150,460, Merck) and a SeQuant^®^ ZIC^®^-pHILIC Guard Kit (20 × 2.1 mm) (50,438, Merck) were used operating at 45 °C. The injection mode was set at 5 μL, and the mobile phase flow rate was set at 0.3 mL/min. Mobile phase solvents were A (95% H_2_O, 5% methanol, 0.1% formic acid) and B (100% methanol). The eluting gradient program in both positive and negative ion mode was the following: 0–1.0 min (95% A, 5% B), 1.0–4.0 min (45% A, 55% B), 4.0–9.0 min (45% A, 55% B), 9.0–10.0 min (20% A, 80% B), 10.0–10.1 min (20% A, 80% B), 10.1–15.0 min (0% A, 100% B), 15.0–15.1min (0% A, 100% B), and 15.1–20.0 min (95% A, 5% B).

#### 2.3.3. Mass Spectrometry

The UHPLC system was coupled to an LTQ-Orbitrap Velos mass spectrometer (Thermo Fisher Scientific, Bremen, Germany) equipped with an APCI source, operating in both positive and negative modes. To monitor the instrument performance over time and chromatographic integrity, including retention time shifts, QC samples were prepared as a mix of each sample. Data were pre-processed with Xcalibur software (version 2.1, Thermo Scientific, Waltham, MA, USA).

#### 2.3.4. Data Processing and Statistical Analysis

Data processing and analysis were performed as described in Chalikiopoulou et al. [31]. ProteoWizard MSConvert [32] was used to centroid all raw MS data and convert them into MzML files prior to MetaboAnalyst 5.0 [33]. LC-MS spectral processing was performed using the auto-optimized parameter setting and blank subtraction. All test-groups were cross-compared, first to gain insights into the GBM metabolomes and then to identify key metabolites. Both positive and negative ion modes were employed during LC-MS analysis. Subsequent analyses included metabolites detected in more than 33% of the samples. Following the removal of uninformative features, the resulting number of metabolites was decreased drastically to ~1/4. For those metabolites surviving our criteria, empty values were annotated with a small value (1). Data centering and unit variance scaling were carried out. Univariate and multivariate statistical analysis were applied where appropriate. Student’s t-test and ANOVA (One-way Analysis of Variance) test followed by post hoc analysis (Fisher’s least significant difference) were used. Critical value was set at <0.05, including FDR correction. For all comparative analysis, we performed Log2fold calculation and PCA and PLS analyses. Next, we determined PLS VIP (variable importance in projection) values. Only metabolites with a log2fold ≥ 2 were selected for subsequent enrichment analysis. Enrichment analysis was performed using Metaboanalyst 5.0 [33] employing pathway-associated metabolite sets (SMPDB). For the interrogation of metabolic pathways, the mummichog algorithm was applied. This algorithm facilitates one-step functional analysis through tandem mass spectra feature tables [34]. The top 10 most significantly associated *m*/*z* features were used as input to the mummichog algorithm v.2. KEGG (Kyoto Encyclopedia of Genes and Genomes) database was selected as the pathway library of interest. Only those metabolic pathways containing at least 3 significant metabolites were included. Significance threshold was set at a *p*-value < 0.05, including FDR correction.

### 2.4. Machine Learning

#### 2.4.1. Supervised Machine Learning

To evaluate the predictive value of those metabolites that have statistically significant differences in their levels between low-risk (OS > 12 months; n = 11) and high-risk (OS < 12 months; n = 10) groups, a discriminant analysis was implemented. A supervised machine learning approach was employed, and hence, several classifiers were tested to find which can model best metabolite levels. Algorithms were trained to stratify patients to either low-risk or high- risk groups based on seven metabolites: perlolyrine, piperidine, hippuric acid, 2,6-diisopropyl-3-methylphenol, dopamine, 7-ketocholesterol, and (±)-(Z)-2-(5-tetradecenyl)cyclobutanone. Metabolites were pre-processed and normalized, before being fed to the classification scheme. To assess the predictive ability of metabolite combinations, Recursive Feature Elimination (RFE) [35] was applied. This feature selection method was evaluated through 10-fold cross validation. Next, four classifiers were trained: SVM [36], Random Forest (RF) [37], eXtreme Gradient Boosting (XGBoost) [38], and Stochastic Gradient Boosting (GBM) [39]. Taking into account sample size and external test feasibility, we opted for a 10-fold cross validation method to evaluate the predictive ability of the algorithms tested as well as the robustness of the models. The metrics that were used for the evaluation of the models were:

$$\mathrm{Accuracy}=\frac{\mathrm{TP}+\mathrm{TN}}{\mathrm{TP}+\mathrm{TN}+\mathrm{FP}+\mathrm{FN}}$$

$$\mathrm{Sensitivity}=\frac{\mathrm{TP}}{\mathrm{TP}+\mathrm{FN}}$$

$$\mathrm{Specificity}=\frac{\mathrm{TN}}{\mathrm{TN}+\mathrm{FP}}$$

$$\mathrm{Matthews}\;\mathrm{Correlation}\;\mathrm{Coefficient}=\frac{\mathrm{TP}\times \mathrm{TN}-\mathrm{FP}\times \mathrm{FN}}{\sqrt{\left(\mathrm{TP}+\mathrm{FP}\right)\left(\mathrm{TP}+\mathrm{FN}\right)\left(\mathrm{TN}+\mathrm{FP}\right)\left(\mathrm{TN}+\mathrm{FN}\right)}}$$

$$F1\;\mathrm{score}=\frac{2\mathrm{TP}}{2\mathrm{TP}+\mathrm{FP}+\mathrm{FN}}$$

where TP indicates the case of a high-risk patient who is correctly classified, and TN denotes the correctly identified low-risk patients by the models. FN corresponds to the case of a high-risk patient who is wrongly predicted as low-risk, and FP denotes the case of a low-risk patient who is classified as high-risk by the models. Next, an analysis of feature importance was implemented to identify which metabolites mostly affect the prediction in question for the model with the optimal performance. The analysis was performed in Rstudio using caret library for machine learning [40].

#### 2.4.2. Unsupervised Machine Learning

An unsupervised method may provide an unbiased indication about whether the integration of miRNA data and metabotypes could have a predictive value. Thus, an unsupervised machine learning approach was also implemented for the validation of the predictive value for the seven metabolites that stratify low- and high-risk patients (perlolyrine, piperidine, hippuric acid, 2,6-diisopropyl-3-methylphenol, dopamine, 7-ketocholesterol, (±)-(Z)-2-(5-tetradecenyl)cyclobutanone). For this, we focused on a cluster analysis for a sub-population of the GBM cohort sharing both miRNA (hsa-miR-20a, hsa-miR-21, hsa-miR-10a) [41] and untargeted metabolomics datasets (n = 7; low-risk, n = 4; high-risk, n = 3). The clustering algorithms used were k-means (k was set to two) [42] and hierarchical clustering [43]. All analyses were carried out with the R programming language.

## 3. Results

### 3.1. Untargeted Metabolomics Suggest Metabolic Remodeling Patterns in GBM Patients with Different Clinical Outcomes and Response to Treatment

The interrogation of GBM plasma metabotypes as well as the informative relationships through which metabolites are connected by our mixed-methods content analysis, followed by queries in the Human Metabolome Database (HMDB) [28], the Metabolomics Workbench, https://www.metabolomicsworkbench.org/ (accessed on 9 October 2020, 9 October 2021 and 29 December 2022) and Metabolights (MTBLS858, MTBLS730, MTBLS3873, MTBLS1558, MTBLS4708) [29] revealed data scarcity and sparsity. There were no available cohorts of low-risk vs. high-risk at diagnosis and during RT + TMZ follow-up neither mass spectrometry-based untargeted metabolomics datasets for plasma. This has been a rather unfortunate outcome despite it adds value to our study presented herein.

Our RT + TMZ patient cohort demonstrated no age- or sex-dependence. Molecular-clinical correlations were drawn to classify low-risk (OS > 12 months) vs. high-risk (OS < 12 months) GBM patients; 52% (n = 11) and 48% (n = 10) were assigned to low- and high-risk groups, respectively.

**Figure 1:**
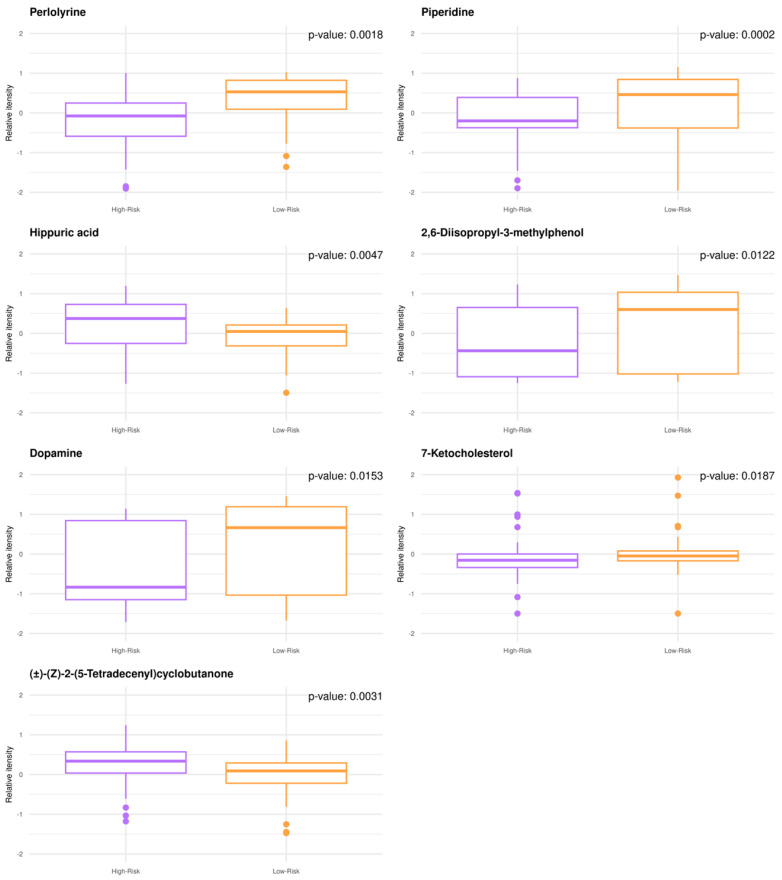
GBM plasma metabotypes enable risk stratification (low-risk, OS > 12 months; high-risk, OS < 12 months). Boxplots of the annotated metabolites with (FC) > 2.0 and *p*-value < 0.05. Individual dots serve as a visual representation of data distribution (such values when inter-individual variability is considered are important features of the data to be analyzed and interpreted). Group comparisons are color-coded: purple, high-risk; orange, low-risk.

Untargeted GBM plasma metabolomics enabled relative quantitative analysis with a high degree of confidence resulting in the annotation and quantification of 1545 metabolites. When comparing the low- to high-risk groups by univariate analysis, n = 38 metabolites were significantly modulated (adjusted *p*-value < 0.05), while n = 30 metabolites exhibited fold change values (FC) > 2.0. The metabotypes of the low-risk patients consist of increased levels of perlolyrine, piperidine, 2,6-disopropyl-3-methylphenol, dopamine, and 7-ketocholesterol, whereas higher levels of (±)-(Z)-2-(5-Tetradecenyl)cyclobutanone and hippuric acid are noted for high-risk patients (Figure 1). Despite being small, significant correlations are found between the known patient characteristics and quantified metabolites.

Additional metabolite changes detected refer to aminoacids, lipids, mitochondrial energy metabolism, and energy substrates. Such metabolic alterations were found when low-risk GBM plasma metabotypes were compared to their high-risk counterparts (group comparison-a) or when baseline GBM plasma metabotypes were compared to those after first RT + TMZ (group comparison-b). For group comparison-a, the most prominent ones, sharing FC > 2.0, yet showing no statistical significance or being statistically significant with FC < 2.0 were: alanine, valine, pyroglutamic acid, 3-methylene-indolenine, and indoxyl. For group comparison-b, acetylphosphate, thymine, histidine, pentadecanoic acid, N-undecanoylglycine, linoleic acid, 3-Methylene-indolenine, tyrosine, and alanylproline were among the most prominent ones.

**Figure 2:**
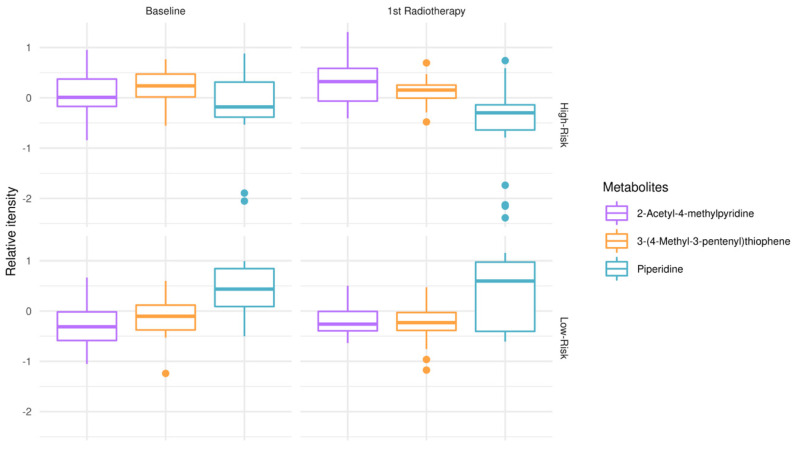
Modulation of metabolites in low-risk patients (OS > 12 months) vs. high-risk (OS < 12 months) GBM patients, at the time of diagnosis (baseline) and after first RT + TMZ. Boxplots of top-metabolites (adjusted *p*-value < 0.05). Individual dots serve as a visual representation of data distribution (such values when inter-individual variability is considered are important features of the data to be analyzed and interpreted). Group comparisons; low-risk patients at the time of diagnosis vs. low-risk patients after first RT + TMZ vs. high-risk patients at the time of diagnosis vs. high-risk patients after first radiotherapy RT + TMZ.

To explore metabolic remodeling patterns when considering response to treatment (RT + TMZ) in addition to clinical outcomes (OS), one-way ANOVA test and post hoc analysis were conducted, comparing low-risk patients at the time of diagnosis vs. low-risk patients after first RT + TMZ vs. high-risk patients at the time of diagnosis vs. high-risk patients after first RT + TMZ (Figure 2). 2-acetyl-4-methylpyridine, piperidine, and 3-(4-methyl-3pentenyl)thiophene were revealed as those significantly modulated among test-groups. Of note, the relative intensity of piperidine appeared to have lower mean values in high-risk patients at the time of diagnosis as well as following first RT + TMZ empowering risk stratification and response to treatment. When interrogating GBM plasma metabotypes during disease progression after therapeutic intervention, independently of risk-groups, there was no statistically significant outcome after false discovery rate (FDR) correction.

### 3.2. GBM Plasma Metabotypes Are Indicative of Disease Severity

**Figure 3:**
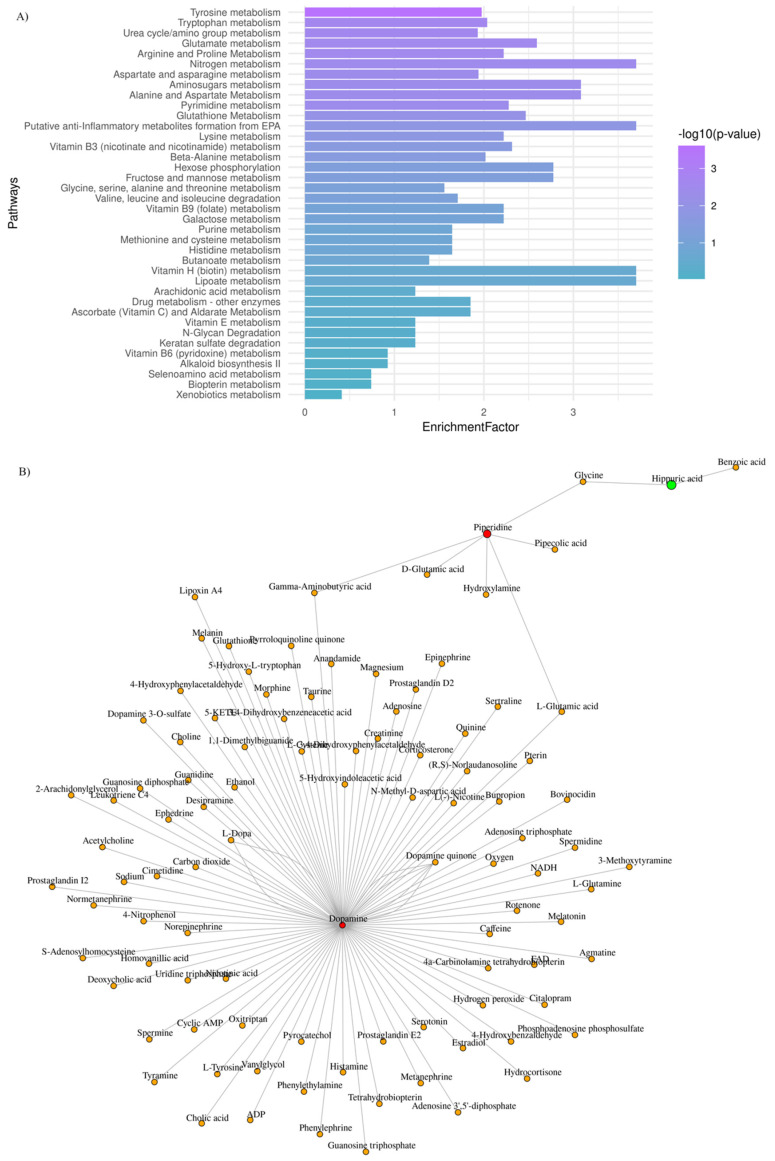
GBM plasma metabotypes reveal metabolic dysregulation that reflects disease severity. (**A**) Mummichog functional analysis was carried out by MetaboAnalyst v.5. Enriched pathways were ranked by significance (see color scale); (**B**) Metabolite-metabolite interaction network of metabolites whose levels are significantly increased (red) and decreased (green), when low-risk vs. high-risk GBM patients are considered.

Aiming to explore further the metabotypes of the low- and high-risk GBM patients, functional enrichment analysis was performed, and the most perturbed metabolic pathways were highlighted (adjusted *p*-value < 0.03; enrichment ratio > 2.0). Data are summarized in Figure 3A.

To draw functional relationships for the test-groups in question, we constructed a subgraph for those metabolites that exhibited (FC) > 2 by querying the KEGG knowledge-based network. As shown in Figure 3B, we identified a subnetwork that consists of dopamine, piperidine, and hippuric acid (99 nodes; 104 edges). Dopamine presented the highest degree value (degree value of 96), which indicates the number of direct neighbors as well as the highest value of betweenness centrality (betweenness of 4717.5), which corresponds to the shortest paths going through this metabolite. Our mixed-methods content analysis was also employed for data interpretation to avoid selection biases.

### 3.3. GBM Plasma Metabotypes Enable Low- and High-Risk Predictions

**Figure 4:**
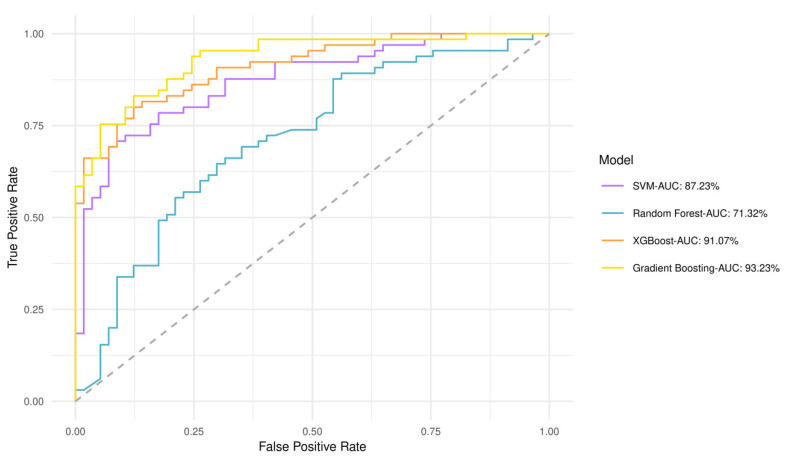
Receiver operating characteristic (ROC) curves and AUCs produced after 10-fold cross validation for the four classifiers trained to stratify low- and high-risk patients based on GBM plasma metabotypes.

A predictive value for perlolyrine, piperidine, hippuric acid, 2,6-diisopropyl-3-methylphenol, dopamine, and (±) -(Z)-2-(5-Tetradecenyl) cyclobutanone was obtained by discriminant analysis based on GBM plasma metabotypes and feature selection analysis (82% accuracy) (Figure S1). Gradient boosting had the best performance and highest area under the curve (AUC) of 93.2%, whereas Random Forest had an AUC of 71.3% (Figure 4). The performance of all models after 10-fold cross validation is provided in Table S2. Gradient boosting had best performance metrics (84% accuracy), reflecting model ability to yield predictions. Matthews Correlation Coefficient suggested that both low- and high-risk patients can be equally predicted. This fact is also evident from the confusion matrix in Table S3, which shows the number of low- and high-risk patients predicted correctly. As shown by the variable importance analysis (Gradient boosting), the contribution of each metabolite in algorithm decisions was also identified (Figure S2). Thus, hippuric acid has the most powerful effect on the model.

Figures S3 and S4 suggest that both unsupervised models (k-means and hierarchical clustering) provide a discrimination accuracy equal to 86%, when miRNA (hsa-miR-20a, hsa-miR-21, hsa-miR-10a) data and metabotypes are integrated. Specificity is equal to 67% and 100% for the low- and high-risk groups, respectively.

## 4. Discussion

Tumor heterogeneity and inter-individual variability are well-established GBM hallmarks, and hence, optimum decision-making post-diagnosis remains a challenge [44]. Deciphering GBM metabotypes as direct indicators of those biochemical changes which define the disease phenotype of an individual may serve as a promising strategy to reveal unique features as well as the mechanistic interplay underlying the observed phenotypic and biological plasticity of GBM [45]. Herein, we employed mass spectrometry-based untargeted plasma metabolomics to gain insights into the rewired metabolic landscape of GBM patients with different clinical outcomes, response to treatment, and disease severity. To avoid selection biases, instead of a population-based estimate, taking into account the sample size of this study and the median OS, 12 months was the cut-off value set for the low- and high-risk groups, as also reported by pivotal studies (RT + TMZ) in GBM and/or clinical trials [46,47,48]. To this end, we also interrogated GBM plasma metabotypes and the informative relationships through which metabolites are connected by a mixed-methods content analysis, followed by queries in the Human Metabolome Database (HMDB) [28], the Metabolomics Workbench, https://www.metabolomicsworkbench.org/ (accessed on 9 October 2020, 9 October 2021 and 29 December 2022) and Metabolights (MTBLS858, MTBLS730, MTBLS3873, MTBLS1558, MTBLS4708) [29]. Data and text mining have been applied in cancer research to facilitate cancer systems biology [49], while Automated Metabolome Assembly has been presented in 2010 as the means to achieve a comprehensive system for metabolome prediction via a text-mining workflow [50].

Piperidine was found to be increased in low-risk GBM patients enabling risk stratification (Figure 1) and was identified among the top 3 metabolites that were significantly altered at the time of diagnosis (baseline) and following the first cycle of radiotherapy(+TMZ) (Figure 1 and Figure 2). This metabolite has been reported in 1977 as a possible neuromodulator in a study by Schmid-Glenewinkel et al. [51] about its biosynthesis by cadaverine and pipecolic acid in mice. The conversion of lysine into piperidine was observed only in the intestines, probably caused by the intestinal flora, while the formation of cadaverine and pipecolic acid from lysine was observed in the brain, liver, kidney, and large intestine. Pipecolic acid was also formed in the heart. In 1983, Nomura et al. [52] suggested that cadaverine is not a precursor of piperidine in brain, the conversion of pipecolic acid into piperidine in the brain does not constitute a major metabolic pathway, and the main source of piperidine in the CNS may be of nonneural origin. Since 1977, the possible contributions of the diet and the intestinal bacteria to the endogenous pool(s) of piperidine have been also discussed. Today, we agree that piperidine—a microbial metabolite—is a naturally occurring metabolite in the human body (a metabolite of cadaverine, a polyamine found in the intestine of humans and mammals) [https://hmdb.ca/metabolites/HMDB0034301(accessed on 9 October 2020, 9 October 2021 and 29 December 2022)].

Our findings agree with Sugimoto et al. who identified piperidine as an oral cancer-specific marker by mass spectrometry-based saliva metabolomics [53]. Saliva is a filtration of blood that can reflect the physiological conditions of the body enabling patient monitoring and the prediction of systemic diseases, while it exhibits diurnal variation and the presence of diverse diagnostic analytes, endogenous plus xenobiotics (similar to blood or urine) [54]. In GBM cell lines, the combination of piperidine or piperidine nitroxide tempol (TPL) with TMZ has resulted in synergistic anti-proliferative action [55]. Of note, a piperidine derivative targeting EZH2 (enhancer of zeste homologue 2) has been reported to reduce GBM cell viability and impair tumor development and aggressiveness via an immunomodulatory mechanism [56]. EZH2 is a S-adenosyl-L-methionine (SAM)-dependent methyltransferase, and its role as an epigenetic modulator in different types of cancer has been widely investigated. In GBM, EZH2 overexpression has been correlated with poor prognosis [57,58]. Although little is known about the mechanistic aspects of EZH2 activity in GBM, the inhibition of EZH2 expression by miR-340 in triple negative breast cancer has led to decreased levels of miR-21, revealing a key miRNA network pathway [59]. In our previous study, we have established a 3-miRNA (hsa-miR-20a, hsa-miR-21, hsa-miR-10a) signature which was able to discriminate low- and high-risk GBM patients, as lower expression levels of miR-21 as well as miR-20a and mir-10a were associated with favorable prognosis [41]. Building on the GBM epigenome-metabolome interplay, we propose a piperidine-EZH2-miR340 mechanistic link—which we have yet to prove.

Taking into account the crosstalk of epigenetic and metabolic signaling in GBM [60], we also noted higher levels of 7-ketocholesterol in the plasma metabolome of low-risk patients (Figure 1). The reprogramming of lipid and cholesterol metabolism has been linked to many cancers, including GBM, while GBM cell growth is highly dependent on cholesterol. Oxysterols, such as 7-ketocholesterol, are oxidized forms of cholesterol that participate in the regulation of cholesterol metabolism through liver X receptors (LXRs) and sterol regulatory element-binding proteins (SREBPs) [61,62,63,64]. SREBPs are highly upregulated in GBM. Furthermore, the feedback loop of miR-29-SCAP/SREBP-1 modulates GBM growth, which is driven by EGFR signaling via the regulation of cholesterol synthesis [65,66]. Oxysterols have been also reported to have antitumor activity in GBM by activating LXRs, thus disrupting cholesterol homeostasis [62]. Our mixed-methods content analysis revealed no output for 2-acetyl-4-methylpyridine, piperidine, and 3-(4-methyl-3pentenyl) thiophene or hippuric acid. For the latter, however, the study of Mallafré-Muro et al. [67] survived some of our multiple levels of interrogation, according to which low levels of hippuric acid are detected in urine samples of colorectal cancer patients.

Among key pathway perturbations, half of the highly enriched and statistically significant pathways relate to amino acid metabolism, namely tryptophan and arginine/proline metabolism (Figure 3). Our findings align with what reported so far. Amino acid metabolism was found disrupted following a nuclear magnetic resonance (NMR)-based metabolomic analysis in plasma samples of glioma patients and healthy controls [68]. The decreased plasma levels of various amino acids in glioma patients may indicate an increased demand for amino acids at the tumor niche [68,69,70]. Synergistically to amino acid metabolism and, in particular, arginine/proline metabolism, the dysregulation of nitrogen and/or pyrimidine metabolism pathways may reflect urea cycle dysregulation, providing essential substrates for tumor proliferation and growth [71,72]. As also suggested by Shen et al. arginine, methionine, and kynurenate were found to be significantly associated with two-year overall and disease-free survival in GBM, indicating the prognostic role of these metabolites [22]. Additionally, glutamine, ornithine, tyrosine, and urea were identified in a serum metabolomic analysis in GBM patients, post-treatment [24].

Interrogating the predictive ability of GBM plasma metabotypes alone (Figure 4) or upon their integration with miRNA data (hsa-miR-20a, hsa-miR-21, hsa-miR-10a) (Figures S3 and S4) [41], both supervised and unsupervised analyses agreed on the predictive value of perlolyrine, piperidine, hippuric acid, 2,6-diisopropyl-3-methylphenol, dopamine, and (±)-(Z)-2-(5-Tetradecenyl) cyclobutanone.

Overall, such key pathway perturbations have been already identified as subjects of epigenetic modulators [60,73]. To our knowledge, only a few studies to date have interrogated GBM plasma metabotypes in well-balanced cohorts at diagnosis and during follow-up (RT + TMZ) by liquid chromatography mass spectrometry (LC-MS), an analytical platform of high sensitivity, as performed herein. Even fewer are those that seek for a combinatorial space of molecular interactions and contexts with emphasis on the epigenome-metabolome interplay. To us, this is where answers to challenging questions are to be found. Why healthy cells acquire and sustain cancer phenotypes? Do canonical driver mutations truly drive tumor development, or do they reflect environmental influences toward clonal selection, and hence, such mutations provide a fitness advantage? At every biological layer investigated so far, cancer cells exhibit dysregulated behavior [74], while emerging datasets suggest that molecular and phenotypic alterations are highly heterogeneous across patients or cancer types or even within the tumor itself [75]. Cancer metabotypes are known to be closer to cellular phenotypes and, thus, provide a more functional understanding of cellular states and transitions. Why? Metabolism is a dynamic process; there is a constant catalysis of metabolic reactions, during which reaction rates and metabolite abundance (intracellular and extracellular) define a metabolic state.

## 5. Conclusions

Our results point to the role of metabolic remodeling in GBM plasticity and disease severity via bioenergetic profiles that map patterns and shed light upon GBM biology and RT + TMZ response. GBM plasma metabotypes alone or upon miRNA data integration are of predictive value, as shown by both supervised and unsupervised analyses. An in-depth understanding of aberrant metabolism promises to provide the framework for personalized metabolic modulation, in particular when the GBM epigenome-metabolome interplay is considered. We envisage that this is how drug repurposing in translational precision medicine will be of benefit [76]: right drug, right dose, right patient (when also timing is right).

## Data Availability

All data generated or analyzed during this study are included in this published article and its supplementary information files.

## Supplementary Materials

The following supporting information can be downloaded at: https://www.mdpi.com/article/10.3390/metabo13030362/s1, Table S1. Demographic and clinical characteristics of the standard-of-care treated GBM patients; Table S2. Performance metrics in 10-fold cross validation process for the four models trained to classify low- and high-risk patients when metabotypes are considered; Table S3. Confusion matrix of each of the classifiers tested (correct and false predictions per model are shown following a 10-fold cross validation); Figure S1. Prediction accuracy during recursive feature elimination process (based on metabotypes); Figure S2. Variable importance analysis (Gradient Boosting); Figure S3. Scatter plot of patients clustered by k-means algorithm based on miRNA expression levels and metabotypes; Figure S4. Hierarchical clustering dendrogram for the low- and high-risk groups based on miRNA expression levels and metabotypes.
