# Supplementary material for: Bioenergetic Profiling in Glioblastoma Multiforme Patients with Different Clinical Outcomes
Source: Metabolites. 2023 Feb 28;13(3):362. doi: 10.3390/metabo13030362 (PMC10051505; doi:10.3390/metabo13030362)
Supplement: Supplementary file 1 [file metabolites-13-00362-s001.zip › metabolites-2224064-supplementary.pdf]

Bioenergetic Profiling in Glioblastoma Multiforme Patients with Different Clinical Outcomes

Vivi Bafiti[1], Sotiris Ouzounis[1], Eleni Siapi[1], Ioanna Maria Grypari[2], Andreas Theophanopoulos[3], Vasilios Panagiotopoulos[3], Vasiliki Zolota[2], Dimitrios Kardamakis[4] and Theodora Katsila[1,*]

Table S1. *Demographic and clinical characteristics of the standard-of-care treated GBM patients*

Demographic/ Clinical Characteristics	GBM cases (n=21)
Sex, Female	12 (55 %)
Sex, Male	9 (45 %)
Race, White	21 (100 %)
Age at surgery/biopsy [years]	56 (28-78)
Overall Survival [months]	12 (2-27)
OS Risk Group, Low Risk Group [OS>12 months]	11 (52 %)
OS Risk Group, High Risk Group [OS<12 months]	10 (48 %)

Table S2. *Performance metrics in 10-fold cross validation process for the four models trained to classify low- and high-risk patients when metabotypes are considered*

	SVM	Random Forest	XGBoost	Gradient Boosting
Accuracy	77.05	82.79	81.15	84.43
Sensitivity	78.43	82.14	80.36	85.19
Specificity	70.18	80.70	78.95	80.70
Matthews Correlation Coefficient	0.54	0.65	0.62	0.69
F1 score	74.07	81.41	79.64	82.88

Table S3. *Confusion matrix of each of the classifiers tested (correct and false predictions per model are shown following a 10-fold cross validation)*

	SVM	Random Forest	XGBoost	Gradient Boosting
True Positive	40	46	45	46
False Negative	11	10	11	8
False Positive	17	11	12	11
True Negative	54	55	54	57

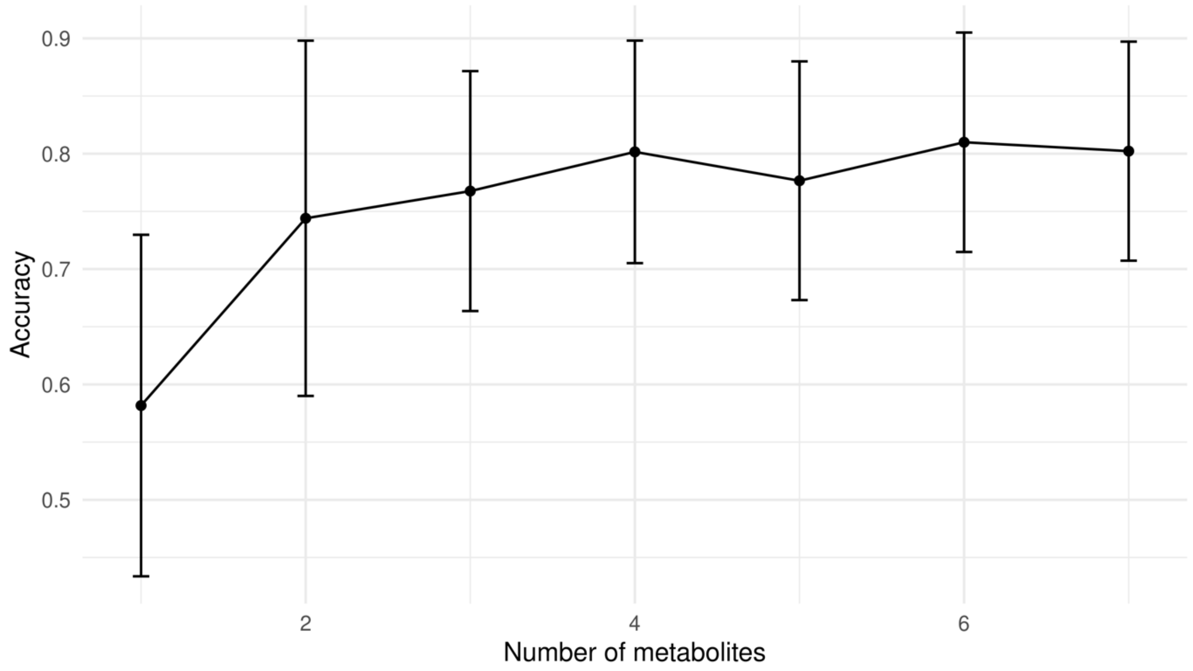

Figure S1. *Prediction accuracy during recursive feature elimination process (based on metabotypes). Error bars indicate the standard deviation of accuracy in 10-fold cross validation*

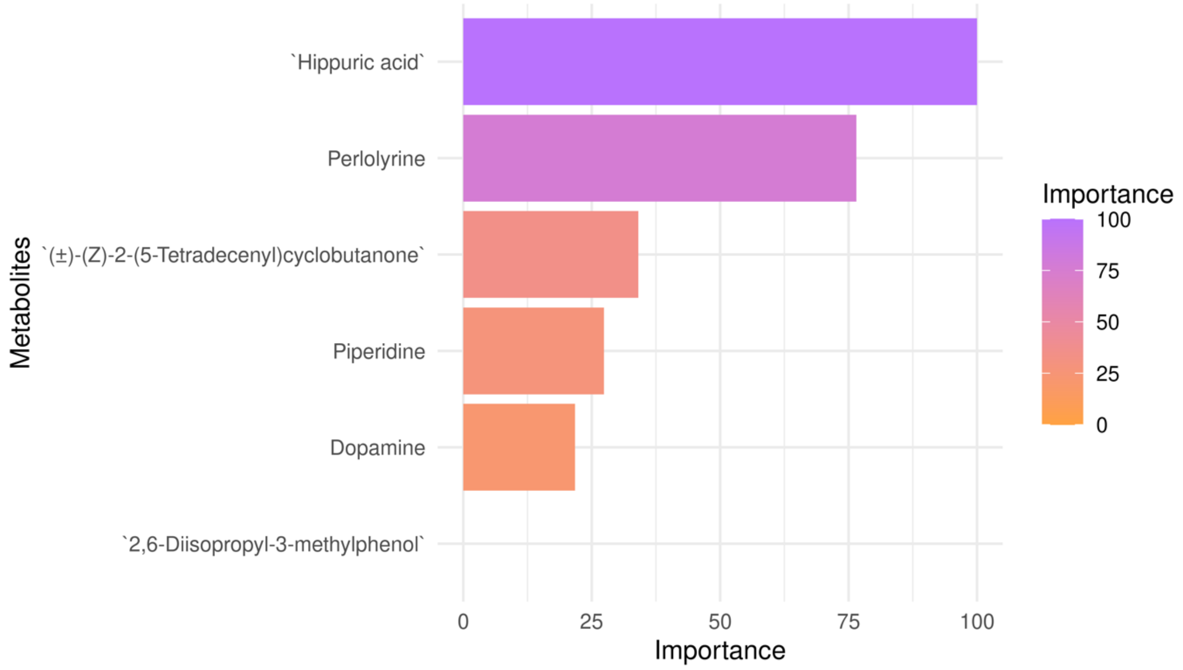

Figure S2. *Variable importance analysis (Gradient Boosting)*

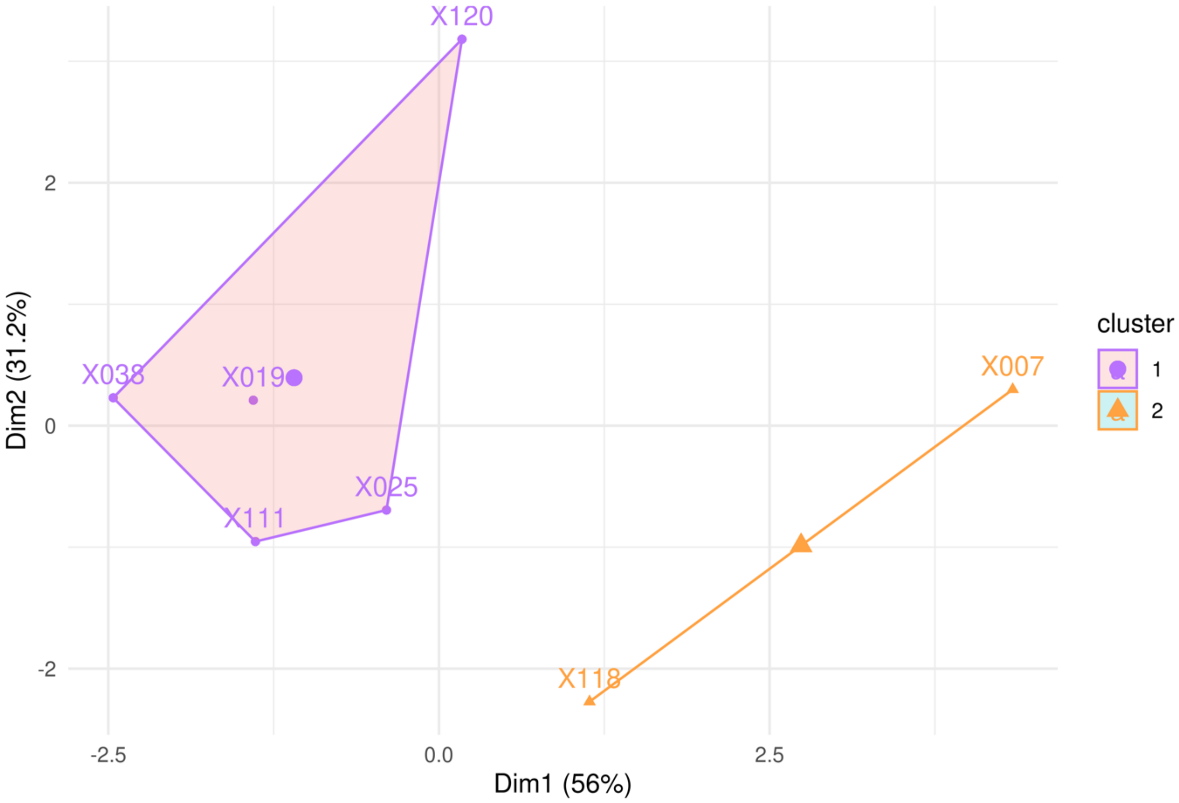

Figure S3. *Scatter plot of patients clustered by k-means algorithm based on miRNA expression levels and metabotypes. Cluster 1, corresponds to the low-risk patients (purple). Cluster 2, indicates the high-risk group (orange). Patient X019 has been grouped to the wrong cluster as dictated by miRNA expression data.*

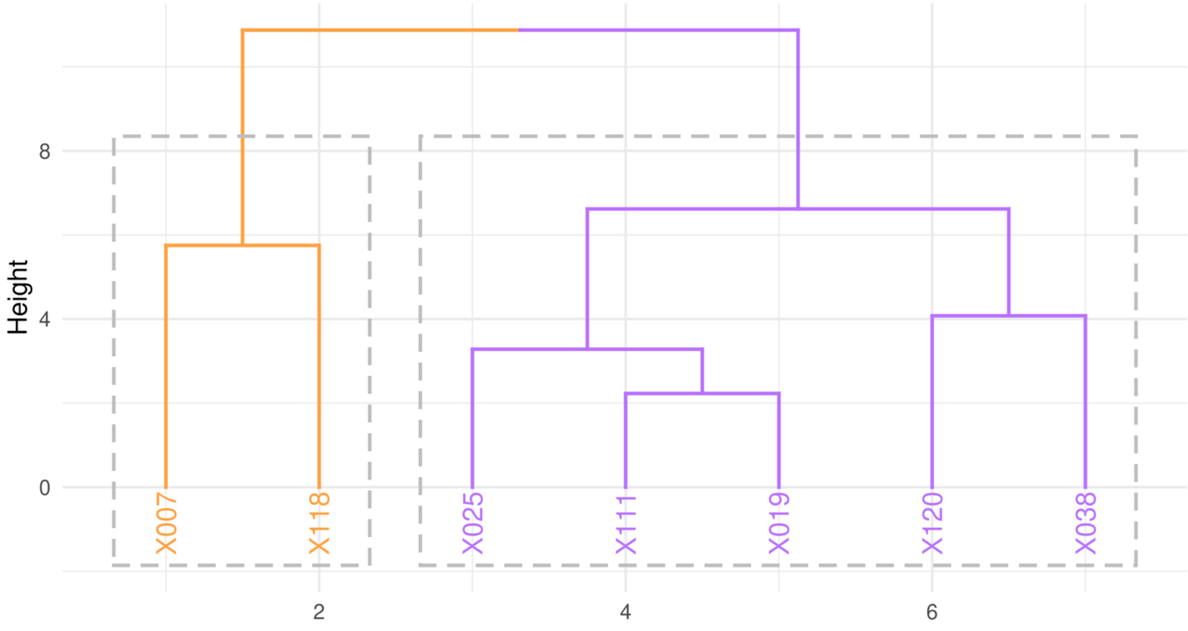

Figure S4. *Hierarchical clustering dendrogram for the low- and high-risk groups based on miRNA expression levels and metabotypes. Low-risk patients are clustered and shown in purple vs. their counterparts (orange). Patient X019 has been grouped to the wrong cluster as dictated by miRNA expression data.*
